# Microcosting Analysis of Diffuse Large B-Cell Lymphoma Treatment in Malawi

**DOI:** 10.1200/JGO.19.00059

**Published:** 2019-07-19

**Authors:** Matthew S. Painschab, Racquel E. Kohler, Edwards Kasonkanji, Takondwa Zuze, Bongani Kaimila, Richard Nyasosela, Ruth Nyirenda, Robert Krysiak, Satish Gopal

**Affiliations:** ^1^ The University of North Carolina Project-Malawi, Lilongwe, Malawi; ^2^ University of North Carolina, Chapel Hill, NC; ^3^ Harvard School of Public Health, Boston, MA; ^4^ Kamuzu Central Hospital, Lilongwe, Malawi; ^5^ University of Malawi College of Medicine, Blantyre, Malawi

## Abstract

**PURPOSE:**

To describe the cost of treating diffuse large B-cell lymphoma (DLBCL) in Malawi under the following circumstances: (1) palliation only, (2) first-line cyclophosphamide, doxorubicin, vincristine, and prednisone (CHOP), (3) salvage etoposide, ifosfamide, and cisplatin (EPIC), and (4) salvage gemcitabine and oxaliplatin (GEMOX).

**METHODS:**

We conducted a microcosting analysis from the health system perspective in the context of a prospective cohort study at a national teaching hospital in Lilongwe, Malawi. Clinical outcomes data were derived from previously published literature from the cohort. Cost data were collected for treatment and 2-year follow-up, reflecting costs incurred by the research institution or referral hospital for goods and services. Costs were collected in Malawian kwacha, inflated and converted to 2017 US dollars.

**RESULTS:**

On a per-patient basis, palliative care alone cost $728 per person. Total costs for first-line treatment with CHOP chemotherapy was $1,844, of which chemotherapy drugs made up 15%. Separate salvage EPIC and GEMOX cost $2,597 and $3,176, respectively. Chemotherapy drugs accounted for 30% of EPIC and 47% of GEMOX.

**CONCLUSION:**

To our knowledge, this is among the first published efforts to characterize detailed costs of cancer treatment in sub-Saharan Africa. The per-patient cost of first-line treatment of DLBCL in Malawi is low relative to high-income countries, suggesting that investments in fixed-duration, curative-intent DLBCL treatment may be attractive in sub-Saharan Africa. Salvage treatment of relapsed/refractory DLBCL costs much more than first-line therapy. Formal cost-effectiveness modeling for CHOP and salvage treatment in the Malawian and other low-resource settings is needed to inform decision makers about optimal use of resources for cancer treatment.

## INTRODUCTION

The majority of cancer deaths now occur in low-income countries.^1^ In sub-Saharan Africa (SSA), an estimated 626,000 people were diagnosed with cancer, and approximately 590,000 died in 2012.^1^ Prospective cancer treatment data from SSA are limited. Furthermore, even if safe and effective, cost considerations for cancer treatments are critical for policymakers in countries where health care resources are highly constrained. Reliable health outcome and economic data are needed for priority setting across competing interests.^2^ However, detailed costs of cancer care in SSA are often not well described. Microcosting, which involves the “direct enumeration and costing of every input consumed in the treatment of a particular patient,”^3(p22)^ provides accurate cost estimates at a programmatic level.

Diffuse large B-cell lymphoma (DLBCL) is the most common non-Hodgkin lymphoma subtype worldwide and in SSA.^4^ DLBCL is curable even in SSA using generic chemotherapy medicines that are typically available in the public sector.^5-8^ We have previously reported our longitudinal experience treating DLBCL in Malawi with cyclophosphamide, doxorubicin, vincristine, and prednisone (CHOP), which is the current regional standard throughout most of SSA.^9^ The 2-year overall survival of DLBCL treated with CHOP in Malawi was 38%.^9^ We report a microcosting analysis using data derived from the same DLBCL cohort receiving routine clinical care at a national teaching hospital to help address the scarcity of detailed, disease-specific cost data for cancer care in SSA to guide regional policymakers.

**CONTEXT**

**Key Objective**
How much does it cost to treat lymphoma in Malawi? Malawi is a low-income country in sub-Saharan Africa. We conducted a microcosting study of cyclophosphamide, doxorubicin, vincristine, and prednisone, and of two salvage chemotherapy regimens in Malawi.
**Knowledge Generated**
First-line treatment with CHOP plus 2 years of follow-up cost $1,844 per patient, on average, much lower than costs in high-income countries. Salvage regimens were more expensive.
**Relevance**
Formal cost-effectiveness modeling is needed to place the cost in the context of clinical efficacy and to help guide policymakers.

## METHODS

### Study Design

We sought to evaluate the cost of first-line therapy of DLBCL in Malawi with CHOP chemotherapy, the current standard of care in the region, as well as two second-line chemotherapy regimens commonly in use on the basis of drug availability and local practice: etoposide, ifosfamide, mesna, prednisone, and cisplatin (EPIC) and gemcitabine and oxaliplatin (GEMOX). We also estimated the costs for palliative care in the setting of relapse or for patients who did not wish to pursue cytotoxic chemotherapy treatment. Up-front palliative care only, without chemotherapy, is largely theoretical because all patients are initially treated with intent to treat with chemotherapy and intent to cure. Each treatment costing and adverse events were considered in isolation from the others. Because the vast majority of health care in Malawi is provided through the public system, the analysis was conducted from a health systems perspective.

### Chemotherapy Treatments

Costs and toxicity data were obtained from the Kamuzu Central Hospital (KCH) Lymphoma Study. The KCH Lymphoma Study is a prospective, observational cohort of pathologically confirmed lymphoproliferative disorders diagnosed and treated at KCH in the capital of Malawi, Lilongwe, as previously described.^10^ After diagnosis, patients underwent comprehensive baseline clinical and laboratory evaluation. First-line treatment was CHOP; second-line treatment of relapsed or refractory DLBCL was a modified EPIC regimen^9,11^ or GEMOX (Table 1). We used data from 86 adult patients enrolled from 2013 to 2017 to estimate real-world data on average chemotherapy use, frequencies of adverse events, and so forth, as described in section Health Events and Outcomes. Over this period, 75 patients from the cohort received first-line CHOP, and 25 patients received EPIC and/or GEMOX for relapsed/refractory DLBCL. Palliative care costs included the costs of diagnosis and supportive care medications to alleviate symptoms. Costs of staging procedures (lumbar puncture and bone marrow biopsy) and laboratory testing needed for the provision of chemotherapy were not included in palliative care costs, because these are not required to provide palliative care alone and would not improve the quality of life of the patient. Of note, all patients underwent staging lumbar puncture, but only patients at high risk for CNS relapse as determined by the CNS International Prognostic Index^12^ were given prophylactic intrathecal (IT) patients received prophylactic IT chemotherapy, or 0.6 doses per patient on average. This was included in the CHOP chemotherapy costs. IT prophylaxis was not routinely given with relapsed/refractory chemotherapy regimens.

**TABLE 1:**
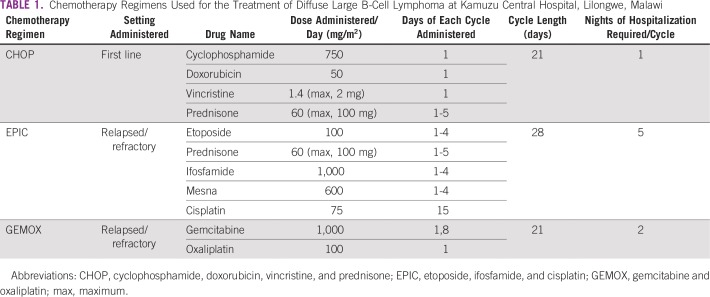
Chemotherapy Regimens Used for the Treatment of Diffuse Large B-Cell Lymphoma at Kamuzu Central Hospital, Lilongwe, Malawi

### Cost Inputs

Data were taken directly from actual costs paid for goods and services at the University of North Carolina (UNC) Project-Malawi and KCH. Variable costs included medications, laboratory tests, transportation reimbursement, clinical and laboratory supplies, radiology testing, and hospitalization costs. Fixed costs included institutional overhead (Appendix Table A1).

Costs were calculated on a per-patient basis for discrete visit/procedure events: tissue biopsy, bone marrow biopsy, lumbar puncture, initial assessment, chemotherapy administration, palliative care, treatment completion, hospitalizations, neutropenic fever, and follow-up. Follow-up was assumed to occur every 3 months after treatment completion for 2 years.

In assigning health care delivery and outcome event probabilities, we used the average number of events over the entire cohort to calculate a per-patient frequency (eg, chemotherapy cycles, hospitalizations, laboratory and radiologic tests) when observational data were available. We made assumptions to estimate frequencies when cohort data were inadequate (Table 2). In these uncertain cases, we sought data from other published sources in Malawi, SSA, or other settings if no local data were available. In all cases, we used the most conservative (ie, most costly) estimates for the base case analysis.

**TABLE 2:**
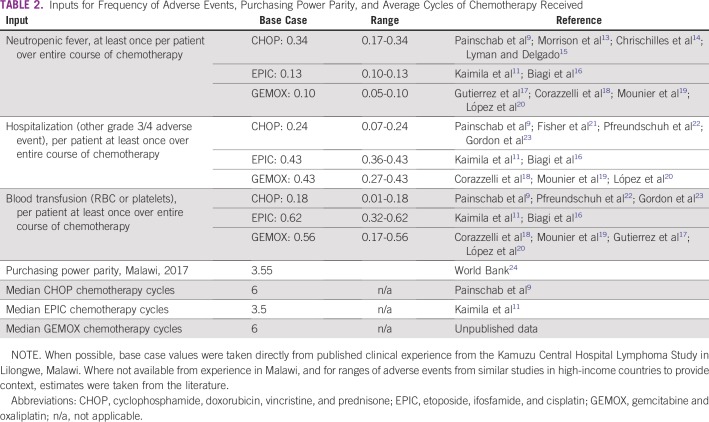
Inputs for Frequency of Adverse Events, Purchasing Power Parity, and Average Cycles of Chemotherapy Received

We conducted interviews with clinical staff and also observed visits/procedures to determine time spent per activity, per patient (eg, chemotherapy administration, bone marrow biopsy). These interviews and time and motion observations were conducted between October 2017 and May 2018 by M.S.P. Time-based activity costs for personnel were calculated based on UNC Project-Malawi salaries.

Overhead costs consisted primarily of pathology and laboratory infrastructure. A large investment of $193,000 was made in 2012 to upgrade pathology and laboratory infrastructure at KCH. We amortized these costs over 25 years. In early experience from the KCH pathology laboratory, 31% of samples were malignancies, and of those, 11% were lymphoma of any kind.^25^ To be conservative, we attributed 5% of laboratory infrastructure costs applied to lymphoma care for the purposes of these analyses. We then applied 4 years of overhead costs (2013 to 2017) over the 86 patients in this series to calculate a per-patient cost of $54.

Patients were treated in the KCH Cancer Clinic at the national teaching hospital, which was in existence before this study and treats patients with cancer referred from the northern and central regions of Malawi. We were not charged for clinic space directly, but rather, estimated physical space costs using typical clinic room rental rates in Lilongwe, equating to $174 per month in 2017 dollars. In reality, this clinic space is used for more than DLBCL treatment, but the full cost was used in this analysis. In addition, we included transportation reimbursement costs for all visits, because these were covered by the research cohort study at $5 in US dollars (USD) per visit, as part of routine program expenses to promote retention.

We adjusted for inflation to 2017 prices using the Malawian gross domestic product deflator.^24^ All costs were converted from Malawian kwacha to USD and international dollars. International dollars were calculated by multiplying the nontradeable goods (blood bank, personnel, hospital stay, transportation) by the purchasing power parity of Malawi in 2017.^24^

### Health Events and Outcomes

The base case of toxicities/adverse events and number of visits for each treatment type were derived directly from clinical trial data from our group in Malawi when available (Table 2).^9,11^ For toxicities that were not available from directly measured data and for ranges of frequencies to provide context, frequencies were derived from the literature.

### Ethical Review

This study was approved by the UNC Institutional Review Board and Malawi National Health Sciences Research Committee. Participants in the clinical studies provided written consent.

## RESULTS

### Microcosting by Episode of Care

Costs were calculated per episode of care as listed in Table 3. We estimated the cost of the initial tissue biopsy to be $115. For patients undergoing treatment, staging included bone marrow biopsy and CNS sampling by lumbar puncture, which cost $92 and $50, respectively.

**TABLE 3:**
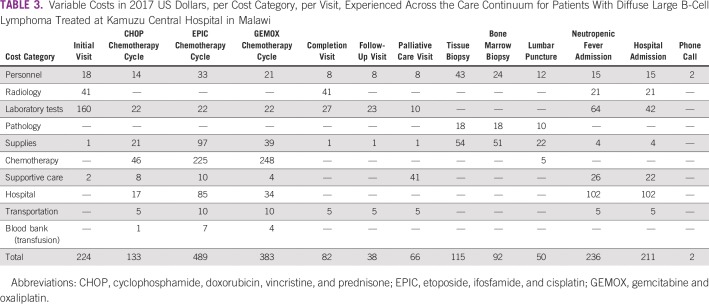
Variable Costs in 2017 US Dollars, per Cost Category, per Visit, Experienced Across the Care Continuum for Patients With Diffuse Large B-Cell Lymphoma Treated at Kamuzu Central Hospital in Malawi

The initial visit, which included basic laboratory examinations, chest radiography, and abdominal ultrasound, cost $224. This was driven largely by laboratory costs, which made up 71% of costs. This encounter type was shared across all treatment comparisons in this study (palliative care, first-line CHOP, and salvage chemotherapy). Each palliative care visit cost $66, largely because of supportive care medication costs (eg, morphine).

**FIG 1:**
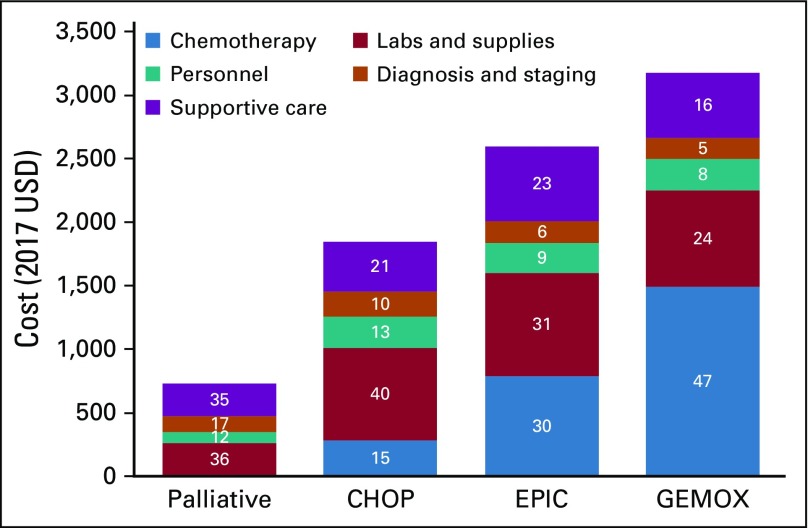
Microcosting analysis of first-line (cyclophosphamide, doxorubicin, vincristine, and prednisone [CHOP]) and second-line (etoposide, ifosfamide, and cisplatin [EPIC] or gemcitabine and oxaliplatin [GEMOX]) treatments for diffuse large B-cell lymphoma in Malawi. Prices in 2017 US dollars (USD) are shown on the *y*-axis. Percentage of total for each cost type is overlaid on the figure.

Chemotherapy costs per cycle varied markedly by regimen (Fig 1). CHOP was $133 per cycle, with drugs accounting for 34% of the per-cycle cost. EPIC and GEMOX were much more: $489 and $383 per cycle, respectively. The chemotherapy drugs alone cost $225 (46%) for EPIC and $248 (65%) for GEMOX per cycle.

All patients completing their course of chemotherapy were expected to return for a completion visit, which we estimated at $82 for a final physical examination, laboratory testing, and radiologic evaluation to assess treatment response. All patients in remission then entered a follow-up phase in which they were seen and evaluated every 3 months through 2 years of follow-up, costing $38 per visit, mostly from laboratory testing.

Costs were also estimated for complications of chemotherapy administration. Each episode of neutropenic fever was estimated to cost $236. Hospitalizations for other serious adverse events were estimated to cost $211 per hospitalization. Blood transfusions cost $42 as provided by the Malawian Ministry of Health.

### Costs by Treatment Type

The mean costs of treating a patient by treatment type are listed in Table 4. Palliative care, which included diagnostic costs, was the least expensive ($728).

**TABLE 4:**
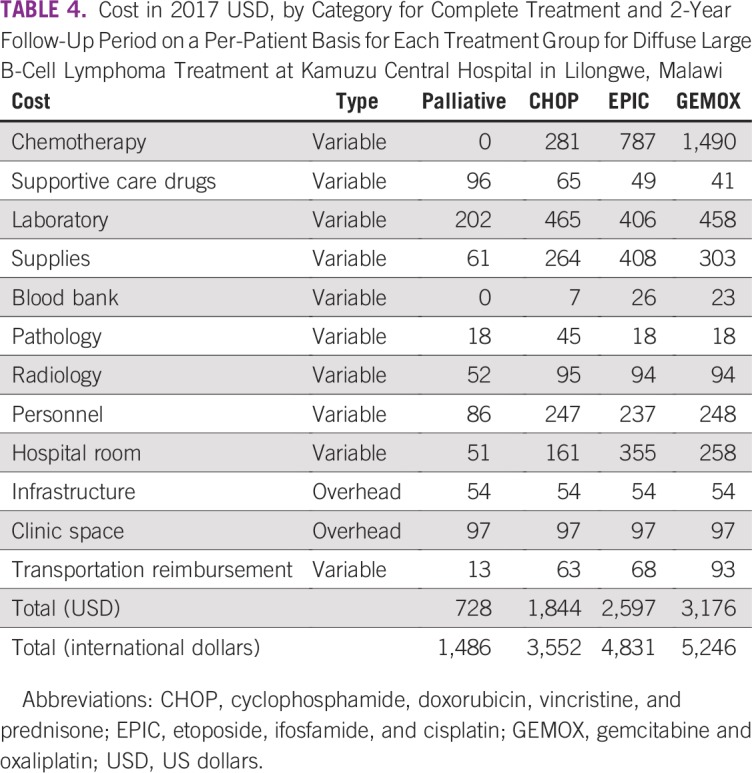
Cost in 2017 USD, by Category for Complete Treatment and 2-Year Follow-Up Period on a Per-Patient Basis for Each Treatment Group for Diffuse Large B-Cell Lymphoma Treatment at Kamuzu Central Hospital in Lilongwe, Malawi

First-line CHOP chemotherapy cost $1,844 per patient. CHOP chemotherapy drugs made up 15% of total costs ($281), whereas laboratory testing made up 25% ($465). EPIC and GEMOX for relapsed/refractory DLBCL were estimated to cost $2,597 and $3,176 per patient respectively. Chemotherapy drugs accounted for 30% ($787) of EPIC, whereas GEMOX chemotherapy was twice as much ($1,490; 47%). EPIC had the highest inpatient hospital charges and supply costs.

## DISCUSSION

In this article, we provide a comprehensive microcosting analysis for DLBCL treatment in Lilongwe, Malawi. We estimated that it cost $1,844 (2017 USD) to treat a patient over the course of 2 years of follow-up with first-line CHOP chemotherapy. Palliative care costs were much lower ($728). However, treatment of relapsed/refractory DLBCL was much costlier: $2,597 for EPIC and $3,176 for GEMOX. The proportion of costs due to chemotherapy drugs varied greatly across treatment types.

In a low-income country such as Malawi, where annual gross national income per capita was $320 in 2017,^24^ $1,844 per patient for first-line DLBCL treatment may seem unaffordable. However, we used conservative assumptions, such as attributing all clinic space to chemotherapy treatment and highest incidence of adverse events, which likely means our cost estimates are high. For example, we used laboratory and personnel expenses incurred at our clinical research program (UNC Project-Malawi), which are significantly more than in the public sector (KCH). Although we did not have access to complete public hospital cost data, public sector laboratory costs are typically substantially lower (approximately half) than what is paid at our clinical research program. In addition, we absorbed the full monthly cost of using the hospital clinic space, not accounting for other types of cancers treated in the same facility.

Chemotherapy drug costs were much lower for first-line CHOP compared with either salvage regimen costs. Chemotherapy costs accounted for nearly half the cost of overall GEMOX treatment compared with 30% for EPIC and 15% for CHOP. As expected, hospitalization and supply costs were highest for EPIC, because of additional overnight stays and additional supplies required with chemotherapy administration. EPIC is given on a 4-day infusion schedule that requires more of these resources than the other chemotherapy regimens, which are delivered over 1 day (CHOP) or 2 days (GEMOX) per cycle. These data demonstrate that even among traditional chemotherapy regimens, wide variations exist in locally incurred costs, which must be considered alongside safety and efficacy considerations in low-resource settings.

Our estimated total per-patient cost of CHOP in this study is much lower than in high-income countries. For example, a US study estimated CHOP treatment and 5-year follow-up to be $7,308 in 2003 USD or $11,694 in 2017 USD, without accounting for treatment-related complications, as we did.^26^ Likewise, an estimate from the French payer perspective, over a 15-year follow-up, was a total cost of $32,524 in 2003 USD or $38,246 in 2017 USD.^27^ The reasons for lower costs in Malawi are likely multifactorial. First, the cost of labor and nontradeable goods are much lower in Malawi as reflected in the purchasing power parity of 3.55 in 2017.^24^ Second, task-shifting strategies and protocol-driven cancer therapy by nononcologist clinicians may be cost saving, as has been shown in other settings and widely adopted for effective scale-up of HIV treatment services throughout SSA.^28,29^ Finally, among other reasons, we hypothesize that price discrimination has allowed low-income countries to acquire drugs and supplies at lower cost.^30,31^ However, this study is not designed to assess which of these mechanisms are most influential.

This study is limited in that the data are largely derived from a single hospital in the context of clinical research activities and thus may not be widely generalizable. Although we are unaware of DLBCL treatment outcomes and economic data from other settings in the region, such data would inform broader regional guidelines with respect to comparative treatment options. However, because we conducted this microcosting study in the context of an observational cohort with comprehensive longitudinal assessment, we captured real-world costs, including those associated with complications and hospitalizations resulting from all treatment.

To our knowledge, this is among the first published efforts to characterize the costs of cancer treatment in SSA. The per-patient cost of first-line CHOP treatment of DLBCL in Malawi is low relative to high-income countries, making it a potentially attractive health investment in SSA in light of the fixed duration of therapy and possibility of long-term event-free survival. However, formal cost-effectiveness modeling for CHOP in the Malawian setting is needed to inform decision makers about treatment program expansion. Salvage treatment costs were much greater than first-line treatment and require formal cost-effectiveness analyses to weigh the costs with the health benefits for relapsed/refractory DLBCL. Modeling would also be helpful to understand the cumulative costing of lymphoma treatment of patients receiving more than one treatment regimen.

## Figures and Tables

**TABLE A1:**
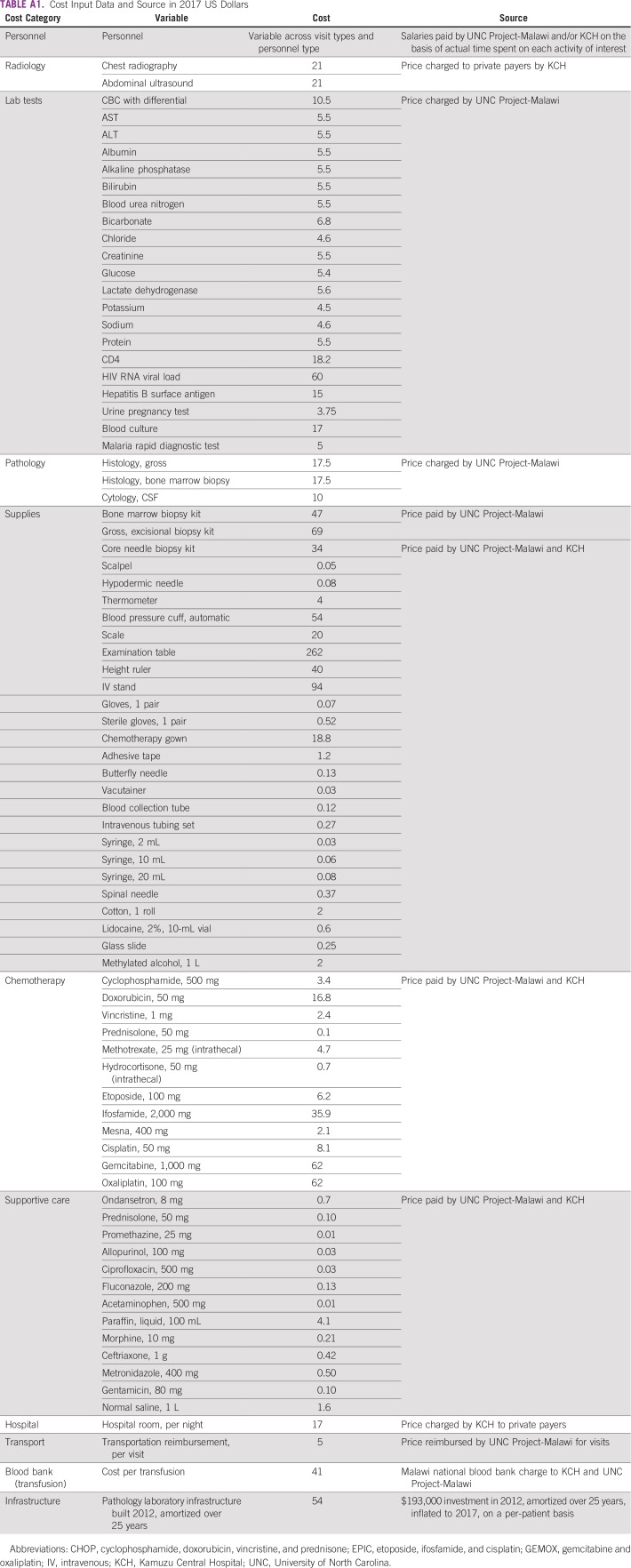
Cost Input Data and Source in 2017 US Dollars
